# Sleeping through the storm: Preventing myelosuppression with quizartinib

**DOI:** 10.18632/oncotarget.21395

**Published:** 2017-09-30

**Authors:** Samuel J. Taylor, Wallace Y. Langdon

**Affiliations:** Wallace Y. Langdon: School of Biomedical Sciences, University of Western Australia, Crawley, Western Australia, Australia

**Keywords:** hematopoietic progenitors, chemotherapy, myelosuppression, FLT3 inhibitors, quizartinib

Chemotherapy-induced myelosuppression is a major complication for cancer patients, causing high rates of morbidity and mortality. Furthermore, myelosuppression is frequently managed by delaying and/or reducing the scheduled dose, and as a consequence the efficacy of treatment can be compromised. In addition, elderly patients are more susceptible to chemotherapy-induced myelosuppression because of their limited hematopoietic reserve, and as a consequence they often receive a reduced dose contributing to poorer outcomes.

Current management of the three main side effects of myelosuppression: anemia, neutropenia and thrombocytopenia is costly and of limited effectiveness. Chemotherapy patients often require hospitalization to treat these side effects, thus affecting their quality of life and causing substantial financial burden to themselves and the healthcare system.

We recently described an innovative approach that prevents chemotherapy-induced myelosuppression in mice through the repurposing of quizartinib, a potent inhibitor of the FLT3 receptor tyrosine kinase [1]. Quizartinib was developed to treat acute myeloid leukemia (AML) patients with FLT3 driver mutations, and clinical trials have shown considerable promise with a high number of complete composite remissions [2, 3]. Our initial studies with quizartinib involved treating *Cbl* RING finger mutant mice that develop a myeloproliferative disease (MPD) [4]. This disease is driven by hyper-active wild type Flt3 signaling [5], and we found that dosing these mice with quizartinib suppressed Flt3 activity, and as a consequence MPD development was prevented [4]. This study was of interest because *CBL* mutations are found in approximately 5% of human MPDs.

**Figure 1:**
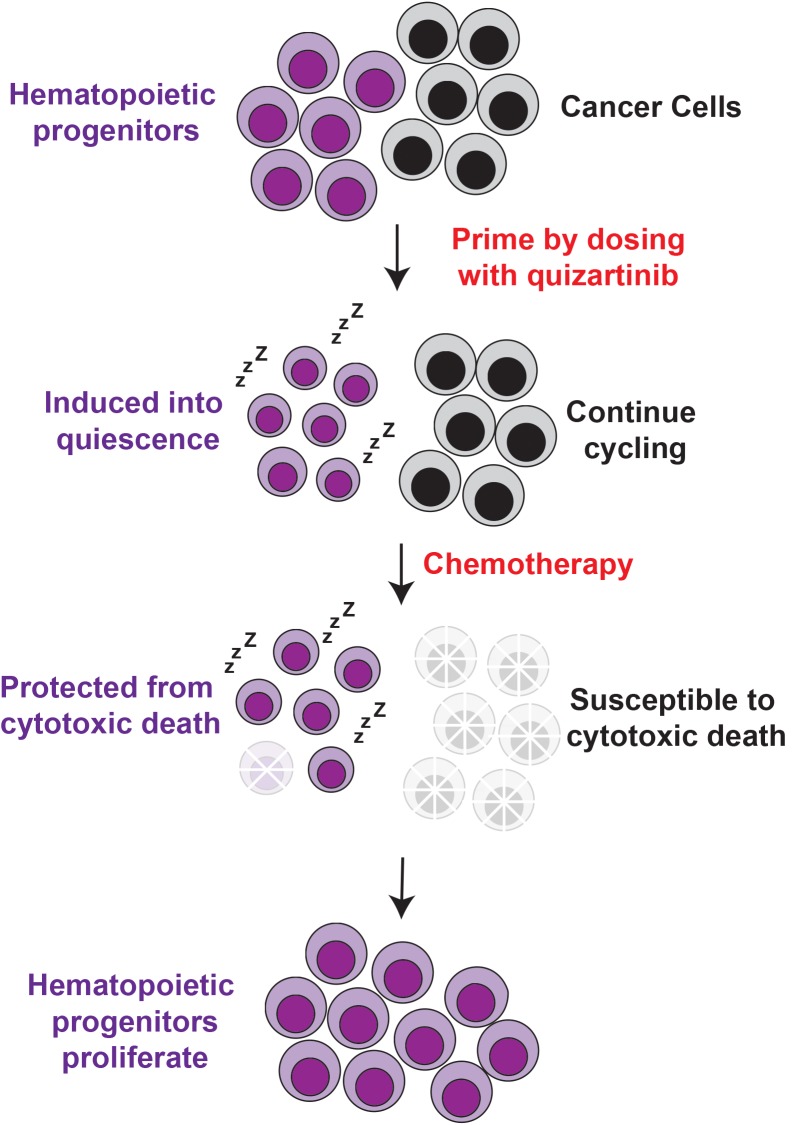
Quizartinib induces transient quiescence in hematopoietic progenitors, but not cancer cells, and this specifically protects the progenitors from chemotherapeutic drugs such as 5-FU and gemcitabine

From these studies of *Cbl* mutant mice we observed that a single dose of quizartinib induced a rapid but transient quiescence in multipotent progenitors (MPPs). MPPs are highly proliferative FLT3^+^ cells in the bone marrow that produce all mature blood lineages. Wild-type C57BL/6 mice were subsequently investigated, and quizartinib produced an identical effect on MPPs as seen in *Cbl* mutant mice. From these observations we reasoned that the transient quiescence might allow the cells to be protected from chemotherapeutic drugs that kill rapidly proliferating cells (Figure 1). A detailed study of doses and time courses revealed that a priming dose of 30 mg/kg of quizartinib 6-18 hours before administration of the cytotoxic drug fluorouracil (5-FU) provided 10-fold protection to MPPs [1]. We also found that quizartinib provided significant protection to committed progenitors within the lineage negative, c-Kit^+^, Sca-1^-^ population (i.e. LK cells), as well more immature CD48^-^ progenitor/stem cells within the lineage negative, c-Kit^+^, Sca-1^+^ population (i.e. LSK cells). As a result quizartinib priming enabled a rapid recovery of bone marrow cellularity after 5-FU treatment, and the numbers of red blood cells, lymphocytes, neutrophils and platelets in the peripheral blood followed a similar trajectory. Furthermore, mice primed with quizartinib before 5-FU retained their body weight, whereas all vehicle-primed mice showed significant weight loss. As a consequence, quizartinib priming prevented otherwise lethal myelosuppression [1] .

We also showed that quizartinib provided marked protection from gemcitabine-induced myelosuppression, and future studies will focus on testing other chemotherapeutic drugs, and combinations, to identify those where quizartinib priming protects the hematopoietic system. It will also be important to determine if additional kinase inhibitors protect the bone marrow from chemotherapy. Recently it was shown that an inhibitor of cyclin-dependent kinases 4 and 6 (CDK4/6), known as G1T28 or trilaciclib, also induced transient quiescence of murine hematopoietic stem and progenitor cells (HSPCs), and protected these cells from 5-FU cytotoxicity [6]. When administered to healthy human volunteers, G1T28 transiently inhibited HSPC proliferation, therefore providing promise for its use in clinical trials. We have found that tyrosine kinase inhibitors dasatinib and imatinib do not induce quiescence, nor protect the hematopoietic system from 5-FU cytotoxicity [1, 7]. Furthermore, crenolanib, another potent FLT3 inhibitor that was developed to treat AML, behaved differently to quizartinib in that it did not induce quiescence of MPPs nor protect them from 5-FU cytotoxicity [1]. It is possible that crenolanib exerts a different biological response because it functions as a type I kinase inhibitor, which binds to an active kinase confirmation, whereas quizartinib, being a type II inhibitor, binds to an inactive kinase conformation.

The protection that quizartinib provides to the hematopoietic system will only be of value to cancer patients if the tumors are not dependent on FLT3 signaling and therefore remain sensitive to chemotherapy. We demonstrated this in two mouse models of AML where the leukemic cells were not induced into quiescence by quizartinib and remained highly sensitive to killing by 5-FU, thus providing a very effective treatment [1]. Clearly, if quizartinib protected the AML cells from the cytotoxic effects of chemotherapy, then the treatment would be adversely affected. Fortunately very few cancers are dependent on FLT3 so it is anticipated that quizartinib priming could be of benefit for the treatment of a wide range of tumors. However, it is likely that this approach could be detrimental for treating disorders such as AMLs with FLT3-ITD mutations. This prediction is supported by an *in vitro* study that found pre-treatment of FLT3-ITD mutant MV4-11 AML cells with the FLT3 inhibitor CEP-701 reduced the number of cycling cells, and as a consequence the cytotoxic effects of cell cycle-dependent chemotherapeutic agents were less pronounced [8].

In summary, the repurposing of quizartinib as a treatment that protects the hematopoietic system from chemotherapy has the potential to transform the management of cancer patients. This approach would not only reduce the debilitating complications associated with myelosuppression, and the considerable expense of its management, it would also protect the immune system’s capacity to fight tumor progression. Since quizartinib is already in use in the clinic it is hoped that this innovative application can soon be trialled in humans.
